# A novel syndrome associated with prenatal fentanyl exposure

**DOI:** 10.1016/j.gimo.2023.100834

**Published:** 2023-09-28

**Authors:** Erin Wadman, Erica Fernandes, Candace Muss, Nina Powell-Hamilton, Monica H. Wojcik, Jill A. Madden, Chrystalle Katte Carreon, Robin D. Clark, Annie Stenftenagel, Kamal Chikalard, Virginia Kimonis, William Brucker, Carolina Alves, Karen W. Gripp

**Affiliations:** 1 Division of Medical Genetics, Nemours Children's Hospital, Wilmington, DE; 2 Division of Newborn Medicine, Boston Children’s Hospital, Boston, MA; 3 The Manton Center for Orphan Disease Research and Division of Genetics and Genomics, Boston Children’s Hospital, Boston, MA; 4 Department of Pathology, Boston Children’s Hospital and Harvard Medical School, Boston, MA; 5 Division of Pediatric Genetics, Loma Linda University Children's Hospital, Loma Linda, CA; 6 Division of Genetics and Genomic Medicine, Department of Pediatrics, University of California-Irvine Medical Center, Irvine, CA; 7 Division of Medical Genetics, Hasbro Children’s Hospital, Providence, RI; 8 FDNA Inc., Sunrise, FL

**Keywords:** Embryopathy, GestaltMatcher, Opioid use disorder, Prenatal fentanyl exposure, Smith-Lemli-Opitz syndrome

## Abstract

A novel syndrome was suspected in individuals sharing short stature, microcephaly, distinctive facial features, and congenital anomalies. We enrolled 6 patients in an institutional review board approved study and evaluated medical history, findings, facial photographs, and test results across this original cohort. Four additional cases with similar findings were contributed by clinicians from outside institutions, bringing the number of reported cases to 10 and supporting the existence of this novel syndrome.

The 6 individuals enrolled into the institutional review board approved study shared microcephaly, short stature, and distinctive facial features. Congenital malformations included cleft palate, talipes equinovarus or rocker bottom feet, and chordee or hypospadias. Short, broad thumbs, single palmar crease, and mild 2,3 toe syndactyly were present. A hypoplastic corpus callosum was noted in 3 of 5 with appropriate evaluation. Their growth and physical findings were suggestive of Smith-Lemli-Opitz syndrome. Biochemical studies shortly after delivery indicated abnormalities in the cholesterol metabolism pathway that subsequently resolved. No shared genomic or genetic cause was identified. All individuals were born after a pregnancy complicated by prenatal exposure to nonprescription opioids, particularly fentanyl, suggesting fentanyl as a teratogen.

Prenatal fentanyl exposure possibly interfered with cholesterol metabolism, giving rise to findings resembling Smith-Lemli-Opitz syndrome. This novel syndrome is clinically recognizable. Four additional cases contributed clinically shared similar findings, increasing the number of cases to 10 and supporting a novel syndrome associated with prenatal fentanyl exposure. Assessment of Shepard and Bradford Hill criteria could be consistent with fentanyl as teratogen, though caution is necessary before assigning causality and data replication is needed.

## Introduction

Identification of a recurrent pattern of congenital anomalies can result in the delineation of a novel syndrome. The definition of a syndrome includes a causality shared among the affected individuals. Exome and genome analysis allow for the efficient identification of shared genetic variants. Novel syndrome delineation now often consists of reviewing individuals ascertained based on their shared genetic variants.^1^ In contrast, embryopathies, such as the recently described congenital Zika syndrome and the more common fetal alcohol syndrome (FAS) or fetal alcohol spectrum disorder, do not have single-gene causes. Here, we report on a cohort of patients, whose physical findings and biochemical abnormalities in the neonatal period suggested a diagnosis of Smith-Lemli-Opitz syndrome (SLOS) (OMIM# 270400), a multiple congenital anomaly syndrome caused by biallelic *DHCR7* variants affecting cholesterol metabolism.^2^ The individuals did not have pathogenic variants consistent with SLOS and did not share other genetic abnormalities. In contrast, all were born after a pregnancy complicated by nonprescription fentanyl exposure, suggesting fentanyl as a possible shared teratogen.

## Patients and Methods

**Figure 1:**
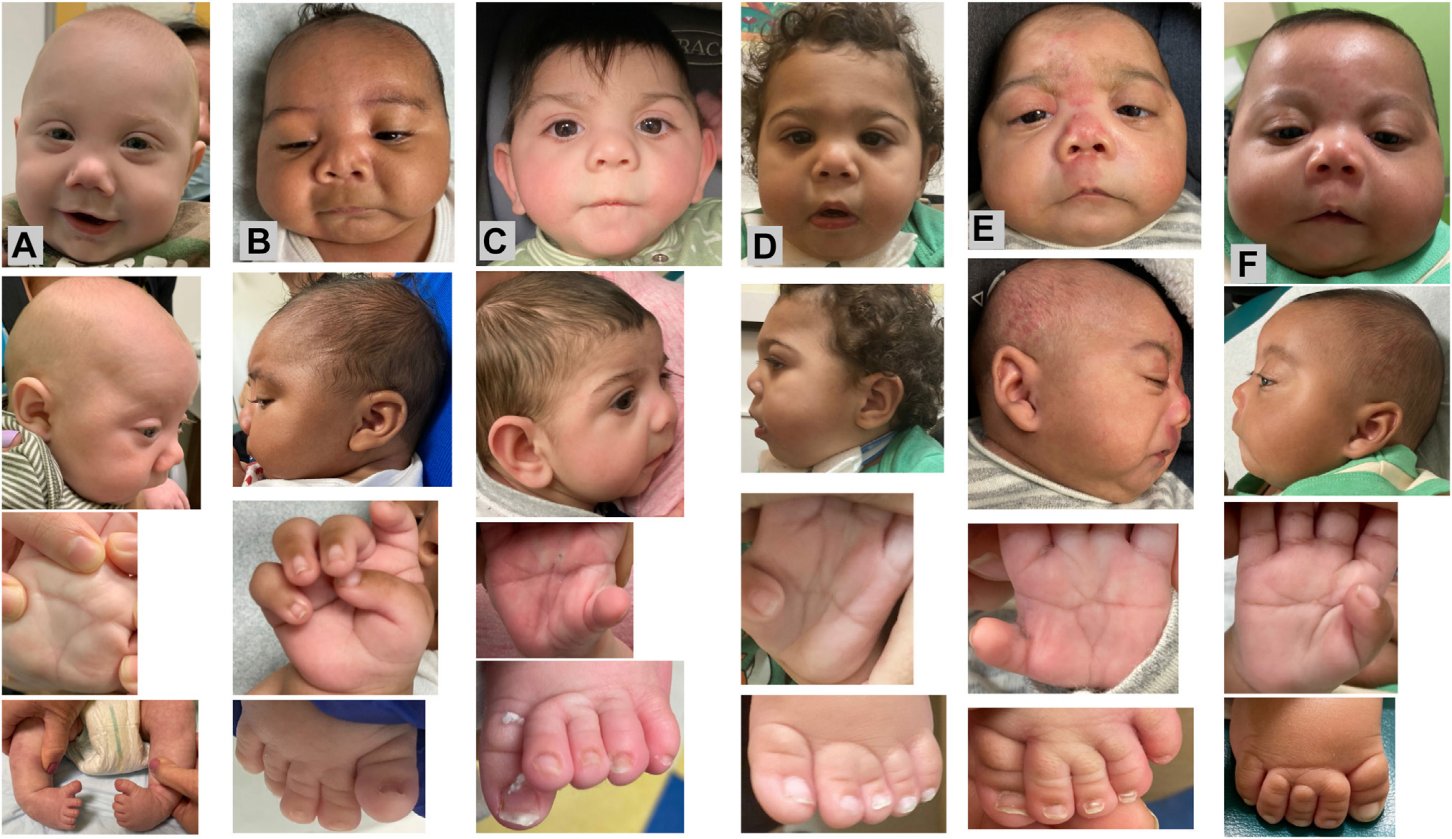
**Facial photographs of Individuals 1-6 (A-F) as used in the GestaltMatcher analysis.** Below each image are a lateral facial view, a hand, and a foot photo of the respective individual.

Clinically identified patients were enrolled into an institutional review board (IRB) approved study and available data were reviewed (Table 1, Supplemental File on Clinical Data). Facial photographs (Figure 1A-F) were analyzed with age matched images from typical individuals (*n* = 10) and those with FAS (*n* = 10) or SLOS (*n* = 9) using Face2Gene’s (FDNA Inc) GestaltMatcher algorithm.^3^

**Table 1:** Clinical information

Individuals	1	2	3	4	5 twin	6 co-twin	Total	Add 1	Add 2	Add 3	Add 4	Total of 10
Sex female (fem), male	male	male	male	male	male	fem	–	male	male	male	fem	–
Prenatal exposures	a, f, h, m	f, o	h, f, o	c, h, f	alc; c, h, f, x		–	cl, f, g, m	f, m	f, m, mar	f, m	–
Gestational age (GA)	36 + 5	37 + 5	39 + 6	39 + 3	34 estimated		–	27	37 + 3	40	39	–
Birthweight g *z*-score for GA	2390 −2.17	3030 −0.67	2640 −2.04	3105 −0.51	1720 −1.3	1600 −1.32	–	865 −0.45	2515 −1.6	4140 1.17	3190 −0.43	–
Length cm *z*-score for GA	45 −2.9	46 −2.05	48 −0.99	49 −0.47	37.5 −2.87	39 −1.89		33 −0.89	40.6 −3	49 −0.96	48 −0.54	–
Head size cm *z*-score for GA	31.5 −2.33	30 −3.52	32.5 −1.54	32.3 −1.7	29 −1.42	26.5 −2.79		23 −1.22	31.5 −2	35 −0.03	33.5 −0.78	–
Short nasal tip	yes	yes	yes	yes	yes	yes	6/6	yes	yes	yes	yes	10/10
Thin upper lip	yes	yes	yes	yes	yes	yes	6/6	yes	yes	yes	no	9/10
Cleft palate	no	yes	yes	yes	yes	yes	5/6	yes	yes	no	no	7/10
Micrognathia	yes	yes	yes	yes	yes	yes	6/6	yes	yes	yes	yes	10/10
Mildly broad, adducted thumb	yes	yes	yes	yes	yes	yes	6/6	yes	yes	no	yes	9/10
Single palmar crease	yes	yes	yes	yes	yes	yes	6/6	yes	yes	yes	yes	10/10
2,3 toe syndactyly	yes	yes	yes	yes	yes	yes	6/6	yes	no	no	yes	8/10
Foot position: talipes (t); rocker bottom (r)	t	no	t	t	r	r	3t 2r	no	r	no	no	3t 3r
Genital anomalies	yes	no	yes	yes	yes	no	4/6	yes	yes	no	no	6/10
Hypoplastic corpus callosum	no	no	yes	yes	yes	na	3/5	yes	no	no	yes	5/9
Elevated 7-DHC or 8-DHC	no	yes	yes	yes	na	na	3/4	yes	yes	yes	yes	7/8
Normal repeat 7- and 8-DHC	na	na	yes	yes	na	na	2/2	yes	yes	yes	yes	6/6
CMA non-diagnostic	yes	yes	yes	yes	yes	yes	5/5	yes	yes	yes	yes	10/10
Exome non-diagnostic	yes	yes	yes	yes	yes	yes	5/5	yes	na	yes	na	7/7
Gene sequence, non-diagnostic	–	–	*DHCR7*	–	na	na	–	*DHCR7*	*DHCR7* Stickler	*DHCR7*	*DHCR7*	–

Prenatal exposures: *a*, amphetamine; *alc*, alcohol; *c*, cocaine; *cl*, clonazepam; *g*, gabapentin; *f*, fentanyl; *h*, heroin; *mar*, marijuana; *m*, methadone; *o*, opiates; *x*, Xanax; *na*: not available or not completed.

Four additional patient reports were contributed with appropriate consent by clinicians from other institutions (Table 1, additional cases, Supplemental File on Clinical Data).

## Results

Six infants (Table 1, individuals 1-6, Supplemental File) were born after pregnancies complicated by multiple drug exposures including fentanyl. The infants had relatively low growth parameters and shared feeding difficulties, distinctive facial features, and physical findings, including single palmar crease and adducted thumb and 2,3 toe syndactyly. Cleft palate (5/6), genital anomalies (4/5 males), and foot position abnormalities (talipes equinovarus in 3 and rocker bottom anomaly in 2) were common. A hypoplastic corpus callosum with diffuse thinning was seen in 3 of 5 who had a brain magnetic resonance imaging study. A clinical suspicion for SLOS led to biochemical testing. Elevated 7-dehydrocholesterol (7-DHC) or 8-dehydrocholesterol (8-DHC) was present shortly after delivery, values normalized subsequently or were normal when testing was performed at age 2.5 months as in individual 1. Chromosome microarray and exome analysis were non-diagnostic.

Analysis of facial photographs (Figure 1, Supplemental Data File on GestaltMatcher analysis) with age matched images from individuals with FAS, SLOS, and typical individuals categorized the novel syndrome as distinct from the other groups. This difference remained highly statistically significant with *P* < .00001 after Bonferroni correction (see Supplemental Data File on GestaltMatcher analysis for details).

Clinicians from other institutions provided information on 4 similar cases of individuals with prenatal fentanyl exposure, abnormal 7-DHC or 8-DHC shortly after delivery with subsequent normalization and non-diagnostic genetic testing (additional cases Table 1, Supplemental File), bringing the total of patients reported here to 10.

## Discussion

Six individuals born after pregnancies complicated by multiple drug exposures, including fentanyl shared physical and facial features suggestive of a novel syndrome. Facial feature analysis using the GestaltMatcher algorithm supported the distinctiveness of the images in this group, compared with images from the considered differential diagnoses of SLOS and FAS, and to typical individuals.

Genetic testing was non-diagnostic regarding a shared underlying cause (Table 1).

### Findings resembling SLOS

The physical findings, including microcephaly and decreased growth, facial features with ptosis, short and anteverted nasal tip, micrognathia, cleft palate, single palmar crease, short thumb, 2,3 toe syndactyly, genital anomalies, and dysgenesis of the corpus callosum resemble those of SLOS.^2^ In the 3 individuals in whom metabolic studies were performed shortly after delivery, abnormalities suggestive of SLOS were noted and resolved subsequently. Individual 3 had biochemical studies demonstrating this time course, with elevated 7- and 8-DHC at age 8 days, normal 7-DHC and less elevated 8-DHC at age 19 days, and normal 7- and 8-DHC at age 5 months. Notably, in individual 1, testing was first performed at age 2.5 months and did not show abnormalities. The elevated 7- and 8-DHC levels are consistent with a disturbance of the cholesterol metabolism pathway prenatally, caused by an extrinsic factor rather than an inborn error of metabolism, allowing for normalization of the metabolic studies in later childhood. The overlap of findings in the novel syndrome with those seen in patients of SLOS supports prenatal cholesterol metabolism abnormalities as the potential cause.

### Drugs interfering with cholesterol metabolism

Psychotropic drugs, such as aripriprazole, haloperidol, and trazodone affect cholesterol metabolism by inhibiting DHCR7, the enzyme catalyzing the final step of the cholesterol metabolism.^4^ In a retrospective study on samples from patients with abnormal 7-DHC levels, exposure to aripriprazole or trazodone was identified in several individuals whose *DHCR7* sequence analysis was negative for SLOS causing variants.^5^

Testing of 727 compounds in the National Institute of Health Clinical Collection identified over 30 compounds that significantly increase 7-DHC; however, there were no opiates studied.^6^ Building upon this work and recognizing the chemical similarities between the DHCR7 inhibiting drugs, computational work identified 3 sets of molecular fragments enriched in the DHCR-7 inhibiting medications.^7^ However, fentanyl and other opioids were not included in this analysis because of their absence in the National Institute of Health Clinical Collection.

Fentanyl is a Sigma-1 protein ligand, similar to cholesterol and many cholesterol precursors. The polycyclic binding pocket of Sigma-1 protein binds most of the inhibitors of the enzymes in the distal half of the cholesterol synthetic pathway (C27 sterols), such as AY9944 and triparanol, the latter bearing some structural resemblance to fentanyl. Because fentanyl is a Sigma-1 ligand, it is reasonable to consider that fentanyl inhibits cholesterol synthesis comparable to AY9944 and triparanol. Early prenatal exposure to fentanyl may affect cholesterol synthesis by inhibition of DHCR7 in the developing fetus, giving rise to physical findings resembling SLOS. Statistical evidence for an increase in malformations in children with prenatal exposure to DHCR7 inhibiting drugs was reported by Boland and Tatonetti, who compared the 10.9 % malformation rate in 321 children born after prenatal exposure to drugs inhibiting DHCR7 with the 0% rate in 612 children with prenatal exposure to the known safe drug levothyroxine, the 3% baseline rate of malformations reported by the Center for Disease Control and Prevention, and a 47.7% rate in 174 children with prenatal teratogen exposure to isotretinoin.^8^ Given the expected 3% baseline rate of malformations, 0% malformation rate after prenatal exposure to levothyroxine is unusual and may suggest an issue with the population studied or the number of births evaluated.

An alternative hypothesis for the teratogenic mechanism is that fentanyl as a Sigma-1 ligand replaces cholesterol in the smoothened receptor’s cholesterol binding pocket, thereby impairing GLI transcription factors activation.^9^

### Prenatal opioid exposure and congenital anomalies

Opioid use in pregnancy and its effect on the fetus have been studied extensively after the increase in prescribed use and associated with opioid use disorder. Preterm delivery, small size for gestational age, reduced head circumference, and sudden infant death, in addition to neonatal abstinence syndrome, can occur in infants with prenatal opioid exposure. Congenital malformations, including cleft lip/palate, talipes equinovarus, genitourinary defects, congenital heart defects, and neural tube defects were seen with an increased frequency in infants with prenatal opioid exposure; however, results varied greatly between studies.^10^^,^^11^ A systematic review noted that 17 of 30 studies with statistical analysis found a positive association between prenatal opioid exposure and oral clefts, ventricular or atrial septal defects, and talipes equinovarus.^11^ Results vary based on cohort size, study design, and selection of the control group, as well as the specific opioid studied. Confounding factors likely play a larger role in illicit use than in prescribed drug use.

Animal models support detrimental effects of prenatal opioid exposure, including decreased birth weight and brain mass and an increase in craniofacial malformation in rats.^12^ Differences in brain development were seen in mice and rats.^12^ An increase in pup mortality occurred in rats.^12^ However, opioids other than fentanyl were used in these animal model studies.^12^ Fentanyl is a highly potent synthetic opioid whose use as an illicitly manufactured substance has increased dramatically. Its transfer to the fetus has been documented in animal models of late pregnancy aiming to reflect the use of fentanyl as analgesic during delivery.^13^ In contrast, transfer in early pregnancy would be implicated in causing structural birth defects, such as cleft palate. Fentanyl transfer across the placenta was documented in human pregnancies scheduled for elective termination between 6 and 16 weeks gestation, thus having the potential to affect the developing fetus.^14^ A similar study on pregnancies between 8 and 14 weeks gestation confirmed these results and found fentanyl in fetal brain tissue, concluding that there is rapid transfer of fentanyl to the fetus in early pregnancy and that the drug remains in fetal tissue for some time.^15^ An in vitro assay of 6 opioids on a human placental trophoblast monolayer found oxycodone to be transferred most efficiently, with fentanyl in an intermediate range and heroin at the lower end. Interestingly, when administered together with fentanyl, the permeability for heroin decreased, whereas fentanyl transfer was not affected by heroin.^16^ This later model may be particularly relevant because fentanyl is likely to be one of multiple compounds consumed in individuals with opioid use disorder. Taken together, these studies show that fentanyl is effectively transferred to the fetus in early pregnancy and thus has the potential to cause developmental defects.

### Shepard and Bradford Hill criteria

Establishing causality for a novel embryopathy may be challenging, as is the case for prenatal fentanyl exposure because, here, the drug is typically used in a non-prescribed and non-documented manner. Shepard’s criteria for teratogenicity in humans can be applied to assess causality as detailed in the Supplemental Material.^17^ Notably, large epidemiologic studies documenting the novel syndrome associated with prenatal fentanyl exposure are not available yet. However, the essential Shepard’s criteria 1 (proven exposure during critical time for development), 3 (careful delineation of clinical cases), and 4 (rare environmental exposure that is associated with a rare defect) may be met and thus causality may be supported.^17^ Similarly, the Bradford Hill criteria can be applied (see Supplementary Material), and 7 of 9 may be considered met currently.^18^ A biologic gradient has not been established, leaving this criterion unmet. The experimental evidence criterion is currently unmet because there is no animal model recapitulating the novel syndrome associated with prenatal fentanyl exposure. Although the 7 other criteria may be met, the evidence remains limited based on the small number of reported patients with this novel syndrome.

### A novel syndrome associated with prenatal fentanyl exposure

The individuals 1-6 were identified based on their distinctive facial features, shared physical findings suggesting a potential diagnosis of SLOS, lack of identifiable genetic cause, and shared history of prenatal drug exposure, including fentanyl. The striking findings in these individuals allowed for the recognition of this novel syndrome. During the process of this study, other patients seen clinically were noted to share some facial and physical features, as well as prenatal fentanyl exposure. However, their findings were less distinctive than those reported here, and we were unable to enroll them into this study. Additional cases (Table 1) were contributed by clinicians from other institutions. We include these cases here because they share prenatal fentanyl exposure, and in each case, effects on the cholesterol metabolism were seen in early screening tests with subsequent resolution. In each additional case, a clinical suspicion for SLOS was raised based on the physical findings. Although these cases share findings with the individuals enrolled in our study, their facial features may subjectively be less striking. Identification and characterization of this novel syndrome associated with prenatal fentanyl exposure will likely result in recognition of less severely affected patients, possible leading to the recognition of a spectrum disorder. Severity of the phenotype may be related to the timing and amount of fentanyl exposure, in addition to possible maternal and fetal pharmacogenomic polymorphisms affecting fentanyl and cholesterol metabolism. In patients with findings consistent with the novel syndrome, or those with a suspected SLOS diagnosis unconfirmed on subsequent testing, maternal use of fentanyl during the pregnancy should be inquired about or documented if information is available.

### Limitations

This report of a novel syndrome associated with prenatal fentanyl exposure has many significant limitations, including the small number of reported individuals, the lack of laboratory studies on fentanyl effects on cholesterol metabolism and fetal development and the absence of an animal model recapitulating the phenotype. Establishing a dose-response relationship between the prenatal fentanyl exposure and the phenotypic findings would support a causal relationship. Unfortunately, because of the difficult social and legal implications of fentanyl use disorder, it is impossible to accurately quantify the timing and amount of prenatal fentanyl exposure. Custody and medical care of the individuals were directly affected by the maternal fentanyl use disorder and data review was difficult because of care being provided in numerous different institutions. The available information remained incomplete and our study approval was limited to review of available records. It is not possible to exclude that a contaminant in the fentanyl, rather than the fentanyl itself, is causal to the phenotype. Nonetheless, this clinical report is critical to delineate this novel condition and to set the stage for future research.

### Future research

This clinical report describes a novel syndrome associated with prenatal fentanyl exposure and recognized in individuals with multiple congenital anomalies and distinctive facial features. The rapid identification of additional cases from other clinicians suggests that this condition is not rare among children born after a pregnancy complicated by maternal fentanyl use disorder. Prenatal exposure to fentanyl should be inquired about in patients with suggestive findings because this information may not be volunteered routinely. The incidence of this novel syndrome needs to be studied, in particular in the context of the fentanyl use epidemic. Bench research into the effects of fentanyl on cholesterol metabolism and embryonic development is necessary to elucidate the mechanism causing the phenotype. Pharmacogenomic studies in the affected patients and their mothers may identify risk factors for the novel syndrome associated with prenatal fentanyl exposure. Long term follow up of patients will delineate possible growth, intellectual and behavioral effects of the condition.

### Conclusions

A novel syndrome, including small size for gestational age, distinctive facial features, cleft palate, single palmar crease, genital anomalies, and 2,3 syndactyly was recognized. When tested in the first weeks after birth, cholesterol metabolism abnormalities suggested SLOS, but genetic testing did not confirm this diagnosis, and subsequent biochemical testing showed a resolution of the abnormalities. No common genetic or genomic abnormality was identified, but prenatal fentanyl exposure was shared among the pregnancies. Although fentanyl’s effect on cholesterol metabolism has not been directly tested, based on indirect evidence it is biologically plausible that it affects cholesterol metabolism in the developing fetus. The additional cases contributed by other clinicians support the causal relationship of the prenatal fentanyl exposure. Despite the limitations of this case report, including the lack of quantifiable prenatal drug exposure and lack of laboratory evidence directly showing fentanyl’s effect on cholesterol metabolism and embryological development, the recognition of the novel syndrome associated with prenatal fentanyl exposure is of critical importance. Future studies are necessary to determine long term outcomes, in particular regarding cognitive abilities and overall wellbeing. In light of the ongoing fentanyl use epidemic, public health impact of the novel syndrome associated with prenatal fentanyl exposure is likely to be significant. Prenatal fentanyl exposure should be inquired about in patients with suggestive findings.

## Data Availability

All data reported in the manuscript and supplemental files.

## Conflict of Interest

Karen W. Gripp is the Chief Medical Officer for FDNA.
