# Supplementary material for: A novel syndrome associated with prenatal fentanyl exposure
Source: Genet Med Open. 2023 Sep 28;1(1):100834. doi: 10.1016/j.gimo.2023.100834 (PMC11613603; doi:10.1016/j.gimo.2023.100834)
Supplement: Supplementary File [file mmc1.pdf]

Supplemental clinical information

Individual 1

This male individual was born at 36 5/7 weeks gestation after a pregnancy complicated by lack of prenatal care and maternal chronic hepatitis C. His mother used nonprescription drugs throughout the entire pregnancy and maternal urine drug screen at delivery was positive for fentanyl, amphetamines and methadone. The mother left the hospital several times against medical advice during labor.

The infant tested positive in urine for fentanyl. He developed neonatal abstinence syndrome requiring drug treatment and prolonged hospitalization.

Newborn hearing screen had an abnormal result. Additional audiometric testing had abnormal results, testing is ongoing and most recently, chronic otitis media was also noted.

At age 4 months his motor development was delayed and he was referred for early intervention services.

Right talipes equinovarus was treated with serial casting.

Genital findings included hooded foreskin and penile torsion.

Brain MRI showed no abnormalities.

Pertinent findings noted on physical exams included distinctive facial features with a short nasal tip; micrognathia; single palmar crease bilaterally; mildly broad and short thumbs bilaterally, spatulate fingertips; broad and short feet.

At age 9 months his weight was 8.8 kg (Z=-0.29); length was 69 cm (Z=-1.8); and head circumference measured at 7 months was 42 cm (Z=- 1.93).

Genetic testing with nondiagnostic results included a chromosome microarray and singleton exome analysis.

Laboratory testing for 7-Dehydrocholesterol was first performed at age 2.5 months and had normal results for 7- and 8-DHC.

Cytomegalovirus PCR testing was performed on the dried newborn screening sample and had a negative result.

Individual 2

This male individual was born after a pregnancy complicated by heavy maternal fentanyl and tobacco use, and limited prenatal care starting at around 32 weeks gestation. The mother used fentanyl throughout the entire pregnancy. Notes from the limited prenatal care included “Patient's medical history is significantly complicated with fentanyl abuse. She has made multiple attempts is (sic) tried to stop cold turkey during this pregnancy, however she was unable to do so. She states that she typically uses 5 bundles of fentanyl day each having 12-13 bags for total of approximately 60 bags of fentanyl daily."

The mother left the hospital three hours after delivery against medical advice.

He has respiratory distress at delivery and required admission to the NICU. He was noted to have dysmorphic features. His meconium drug test was positive for morphine. He had significant neonatal abstinence syndrome (NAS) managed with morphine for 23 days. Additional findings included microcephaly, 2/3 toe syndactyly and abnormal foot position, as well as a cleft palate. MRI brain was limited, but showed absent septum pellucidum and right retinal coloboma. Ophthalmology evaluation noted bilateral retinal colobomata, smaller on the left than on the right.

A G-tube as placed for persistent feeding difficulties.

The cleft palate was surgically repaired at age 12 months.

Bilateral calcaneovalgus position of his feet and adduction contractures of his hands were present. The foot position was treated with physical therapy and braces were prescribed at age 10 months.

High muscle tone in his extremities persisted and was treated with gabapentin at age 1 year.

Unilateral inguinal hernia was surgically repaired.

At age 13 months his weight was 8 kg (Z=-1.95); length was 64 cm (Z=-5.32); and head circumference measured at 12 months was 42.5 cm (Z=- 2.83).

At age 5 weeks, his 8-DHC was minimally elevated and 7-DHC was in the normal range.

Chromosome microarray showed a 482 kb deletion of 3p24.3, which was classified as a variant of uncertain significance (VUS), as there were no clinically relevant genes in the deletion (arr[GRCh37] 3p24.3(19158018-19639711)x1) .

Singleton exome analysis identified a VUS in DCX (c.800 A>G; p.(D267G)).

Individual 3

This boy was born at full term after a pregnancy complicated by late prenatal care. Maternal substance abuse including heroin, opiates and fentanyl was reported throughout the entire pregnancy. An attempt at a buprenorphine/naloxone program was unsuccessful. Maternal urine drug screen at delivery was positive for fentanyl. Individual 3 was small for gestational age and had multiple malformations including micrognathia, cleft palate, hypospadias with severe ventral chordee, cryptorchidism, bilateral talipes equinovarus, bilateral 2/3 toe syndactyly and dysmorphic facial features. SLO was suspected and biochemical testing was completed on DOL 8 with abnormal results (7-DHC 6.9 (normal <2.0mg/L); 8-DHC 14.2 (normal <0.3mg/L)).

Persistent feeding difficulties required placement of a G-tube.

Cleft palate was surgically repaired at age 11 months.

Audiology evaluation identified moderate to moderately severe sensorineural hearing loss in the right and severe hearing loss in the left ear, treated with hearing aids bilaterally.

Brain MRI identified mild hypoplasia of the splenium of the corpus callosum; and internal auditory canal MRI showed unremarkable structures.

At age 13 months his weight was 6.28 kg (Z=-4.10); length was 64.5 cm (Z=-5.15); and head circumference measured at 11 months was 42 cm (Z=- 2.95).

Results of biochemical testing:

DOL 8 with abnormal results (7-DHC 6.9 (normal <2.0mg/L); 8-DHC 14.2 (normal <0.3mg/L)).

DOL 19 with abnormal results (7-DHC 0.7 (normal <2.0mg/L); 8-DHC 7.4 (normal <0.3mg/L)).

At age 5 months, normal results (7-DHC 0.3 (normal <2.0mg/L); 8-DHC 0.1 (normal <0.3mg/L)).

Chromosome microarray had a normal result, as did sequencing and deletion/duplication studies on *DHCR7*. Trio exome analysis with mitochondrial genome showed no reportable variants.

Individual 4

This boy was born at 39 3/7 weeks gestation after a pregnancy complicated by substance abuse including heroin, cocaine and fentanyl, late prenatal care and maternal hepatitis C. Meconium drug screen was positive and additional chromatography/mass spectrometry confirmed positive results for amphetamines, barbiturates, benzodiazepines, cocaine metabolites, marijuana metabolites, opiates, methadone, phencyclidine and propoxyphene.

Notable physical findings included distinctive facial features with short nasal tip; cleft palate; micrognathia; short and adducted thumbs; single palmar creases; bilateral 2,3 toe syndactyly. Hypospadias with chordee and penile web was noted

A tracheostomy was placed due to respiratory distress and abnormal vocal cord placement. G-tube placement was required due to persistent feeding difficulties.

Pulmonary hypertension required medication treatment.

A right talipes equinovarus was treated with serial casting.

Echocardiogram showed a patent ductus arteriosus and an ASD.

Brain MRI showed mild hypoplasia of corpus callosum and bilateral frontal lobes. The intraventricular septum was not identified.

At age 24 months his weight was 9.6 kg (Z=-2.67); length was 74.9 cm (Z=-3.55); and head circumference measured at 22 months was 41.9 cm (Z=- 4.66).

Biochemical testing on DOL 8 had abnormal results (7-DHC 6.1 (normal <2.0mg/L); 8-DHC 10.5 (normal <0.3mg/L)). At age 6 months, 7- and 8-DHC levels were normal.

DNA sequencing of *DHCR7* was negative for pathogenic variants.

He had normal results for chromosome microarray. Singleton exome analysis was non diagnostic, but identified a VUS in *FRMPD4* (c.221C>T; p.(Pro74Leu)) (NM_014728.3).

Individual 5

This male was born after a twin pregnancy with no prenatal care and uncertain gestational age. The mother was 36 years old at delivery. The pregnancy was complicated by self-reported maternal use of heroin, xanax, cocaine and fentanyl, as well as daily cigarette smoking and alcohol consumption about twice per month. Though it was unclear when the pregnancy was conceived, the mother reported continued drug use throughout the entire pregnancy. Intranasal use of fentanyl, heroin and cocaine was reported on the day of delivery.

Delivery occurred by c/section when the mother presented in preterm labor.

Newborn hearing screen and follow up testing was abnormal. Audiology evaluation showed results consistent with a "possible greater than mild deficit in the cochlear component of the auditory system bilaterally".

A gastrostomy tube (G-tube) was placed for persistent feeding difficulties.

Genital findings as described by urology were "significant penoscrotal webbing with very short ventral skin, slight chordee due to skin tethering, buried shaft".

Bilateral oblique talus foot position presented with bilateral rocker bottom foot appearance. Both feet required serial casting for correction.

Pertinent findings on physical exams included microcephaly; capillary nevus over the glabella and face; short nasal tip; cleft palate; low-set ears; micrognathia; single palmar crease and adducted thumbs bilaterally; rocker bottom feet; 2,3 toe syndactyly.

Brain MRI showed mild dysgenesis of the corpus callosum.

Echocardiogram had essentially normal results.

At age 5 months (adjusted age 4 months), his weight was 5.5 kg (adjusted for GA, Z=-1.59); length at age 4 months was 50 cm (adjusted for GA, Z=-4.92) and head circumference measured at 4 months was 36.4 cm (adjusted for GA, Z=- 2.98).

Genetic testing included a normal result on chromosome microarray and no reported findings on exome analysis (including sample from mother and co-twin Individual 6).

Individual 6

This female individual was the co-twin to Individual 5.

She was hospitalized for 60 days due to preterm delivery and small-for-gestational age. Hyperbilirubinemia due to ABO blood group incompatibility did not require phototherapy. Perinatal hepatitis C and HIV exposure was treated. Neonatal abstinence syndrome did not require treatment.

An echocardiogram had an essentially normal result. She required G-tube placement on DOL 54 for persistent feeding difficulties. A brain MRI has not been completed.

Pertinent positive findings on physical exams included: Microcephaly; micrognathia; short nasal tip; submucous cleft palate with bifid uvula and palpable posterior palatal notch; low-set ears; short neck with webbing; bilateral rocker bottom feet; single palmar crease bilaterally; short and adducted thumbs;

At age 6 months (adjusted age 5 months), she showed motor delay with being unable to roll over.

Weight was 5.2 kg (adjusted for GA, Z=-2.25). Length measure 50.8 cm (adjusted for GA, Z= -5.94). Head circumference was 36.3 cm (adjusted for GA, Z= -4.03).

Genetic testing included a normal result on microarray and no reported findings on an exome analysis performed in combination with samples from the co-twin (Individual 5) and the mother.

<u>Additional cases provided from clinicians at other institutions with appropriate consent</u>

Additional Case 1

This male infant was born at 27 weeks gestational age to a mother with opioid use disorder, reportedly in remission and on methadone during the pregnancy, with delivery in the context of a fentanyl overdose. Prenatal imaging was reportedly normal aside from growth restriction. Prenatal drug exposures included clonazepam and gabapentin, in addition to methadone and fentanyl.

He required intubation after delivery and continued to be ventilator dependent. The individual was on opioid infusions for several weeks after delivery due to critical illness, ultimately transitioning to enteral opioids until the time of death.

The prominent features for the individual included SGA, hypertelorism, downslanting palpebral fissures, epicanthal folds, anteverted nares, flat nasal bridge, thin vermillion, cleft palate, redundant nuchal tissue, secundum-type atrial septal defect with mildly reduced LV cavity chamber size and mitral and aortic valve annulus diameters in addition to multiple blood cysts on the tricuspid and mitral valve leaflets, bilateral 2,3 syndactyly of the toes, single palmar creases, small phallus, bilateral undescended testes and a bifid scrotum.

Brain MRI showed a hypoplastic corpus callosum.

Echocardiogram showed pulmonary vein stenosis and a secundum ASD.

The infant died at age 3 months after a complicated NICU course and an autopsy was performed. CNS findings on autopsy were notable for microcephaly, cerebellar hypoplasia, and right hippocampal

"caterpillar" malformation, all thought to be consistent with SLO. A valgus deformity of the feet was noted.

At age 3 months, his length was 45 cm (Z=-3.03 corrected for postmenstrual age); and his weight was 2.92 kg (Z=-1.07 corrected for postmenstrual age); his head circumference measured 29 cm (Z=-4.83, corrected for postmenstrual age).

On DOL 2, he had elevated 7-DHC (68 ug/ml) and 8-DHC (33 ug/ml) and very low total cholesterol (19 mg/dl).

Repeat testing at age 2.5 months had slightly elevated 7-DHC (0.46 ug/mL - mildly elevated but not in SLO range) and normal 8-DHC results.

Another repeat at age 3 months had similar results (7-DHC 0.44 ug/mL). Both follow-up cholesterol panels were drawn when the infant was on cholesterol supplementation and on continued opioids.

Genetic testing with non-diagnostic results included karyotype, *DHCR7* sequencing and del/dup analysis, trio exome analysis, and genome sequencing with RNA sequencing. Chromosome microarray was non-diagnostic with 2 VUS (0.227 MB gain on 15q13.3; 0.202 MB gain on 6p22.1).

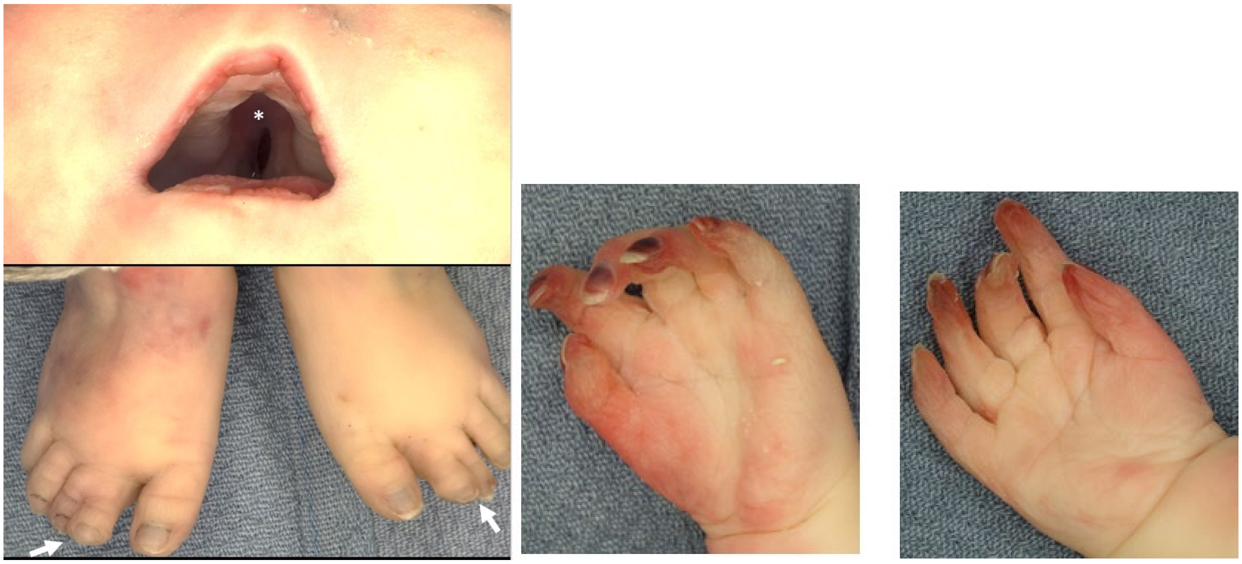

Post mortem images of additional Case 1, showing thin vermillion and cleft palate (upper) and bilateral 2,3 toe syndactyly (lower image); single palmar creases and short adducted thumbs (middle and right image).

Additional Case 2

This male individual was born after a pregnancy complicated by reported fentanyl use in the first months of pregnancy. Maternal methadone use was continued throughout the pregnancy. A maternal urine drug screen during the pregnancy was positive for amphetamines. Maternal urine drug screen at delivery was negative.

Fetal ultrasound reported short long bones, however, the study was deemed suboptimal due to late gestational age and fetal position.

Physical findings described postnatally included cleft palate requiring surgical repair; micrognathia; genital abnormalities including hypospadias and chordee; and rocker bottom feet.

On dysmorphology exam, facies suggested SLO with flat facies, shallow orbits, mild hypertelorism, bilateral telecanthus, infraorbital creases, depressed nasal bridge, anteverted nasal tip, short nose with long philtrum, milia on nose and micrognathia. Cleft palate, broad neck with excess posterior nuchal skin, bilateral single palmar crease, hypoplastic nails and clinodactyly of both little fingers, adducted thumbs, generalized hypertonia, and shrill high pitched cry were noted.

Brain MRI was reported as normal.

At age 6 months, his development was considered appropriate for age 4 months, with smiling socially, tracking with his eyes, grasping for objects and rolling over.

At age 9 months, his height was 60 cm (Z= -5.23); weight 6.515 kg (Z= -2.79); head circumference was 42 cm (Z= -2.51).

Biochemical studies:

DOL 0 with abnormal results (7-DHC 6.5 (normal <2.0mg/L); 8-DHC 8.5 (normal <0.3mg/L)).

DOL 134 with normal results (7-DHC 0.3 (normal <2.0mg/L); 8-DHC 0.1 (normal <0.3mg/L)).

Genetic testing included DHCR7 and Stickler gene panel analysis; chromosome microarray was non diagnostic.

Additional Case 3

This male patient was delivered by C-section at 40 weeks gestation to a 28-year-old G2P2 mother with the prenatal history of maternal opioid use disorder and a positive maternal urine drug screen (fentanyl, methadone, marijuana) during third trimester. He had neonatal abstinence syndrome and persistent feeding difficulties. Dysmorphic features included ocular hypertelorism, downslanting palpebral fissures, midface hypoplasia, flat and broad nasal bridge, nuchal redundancy, micro and retrognathia, and transverse palmar crease. Echocardiogram showed a patent foramen ovale. His brain MRI showed mild sylvian cisterns that suggested some underdevelopment of the brain overall for age.

His postnatal course was significant for G-tube dependent feeding, microcephaly (Z= -3.32), and poor weight gain (Z =-2.95) with more normal linear growth (Z= -1.52). He had diffuse hypotonia and developmental delay but continued to make developmental progress and did not have epilepsy.

He had a 16-months-older full sibling older who also had prenatal fentanyl exposure with a similar phenotype including microcephaly, g-tube dependent feeding and significant developmental delays. No biochemical testing was completed on this sibling.

Biochemical testing on DOL 7 had abnormal results: 7-dehydrocholesterol: 20.5 mg/dL (normal < 2.0); 8-dehydrocholesterol 37.7 mg/dL (normal < 0.3). He was started on cholesterol supplementation when

the abnormal sterol metabolism results were identified and received 250 mg cholesterol twice daily for 4 months. Repeat biochemical testing on DOL 30 had normalized 7-dehydrocholesterol (0.7 mg/L) and decreased but persistently elevated 8-dehydrocholesterol (4.4 mg/L). Genetic testing for DHCR7 sequence variants was negative and subsequent biochemical testing with an expanded plasma sterols panel was normal.

Chromosomal microarray identified a maternally inherited 225 kb deletion of uncertain significance at 2q22.1.

Exome sequencing was nondiagnostic.

Mitochondrial sequencing identified a maternally inherited mitochondrial VUS (m.8707 C>T, p.His61Tyr) at 2% heteroplasmy.

Additional Case 4

This female patient was born via C-section at term to a 23-year-old mother with pregnancy complicated by methamphetamine use during the first 2 months, and fentanyl use throughout pregnancy. Physical exam was significant for mildly low-set dysplastic ears, almond-shaped eyes bilateral 2,3-toe syndactyly, bilateral single palmar crease, and a slight increase in nuchal skin folds. She underwent management for neonatal abstinence syndrome, but continued to have difficulty feeding post-withdrawal, eventually requiring PEG placement.

She had agenesis of the corpus callosum and bilateral optic nerve hypoplasia with unclear visual prognosis.

A developmental assessment performed at age 7 months using the Bayley-4 tool showed cognitive development at the 50th centile; language composite score at the 23rd centile; motor composite score at the 19th centile; sensory processing skills and oral feeding skills were age appropriate.

At age 8 months, her weight was 6.74 kg (Z= -1.58); length 62.4 cm (Z= -3.06) and head circumference was 40 cm (Z= -2.77).

Screening for SLO on DOL 6 found elevated 7-DHC (2.5 mg/L, Normal ≤ 2.0) and 8-DHC (8.2 mg/L, Normal ≤ 0.3). Subsequent testing 1 month later sent to the specialty laboratory showed normal levels of 7-and 8-dehydrocholesterol. Confirmatory genetic testing for *DHCR7* pathogenic variants was negative. Microarray testing demonstrated proximal duplication (BP2 to BP3 region) of 1q21.1 of undetermined significance.

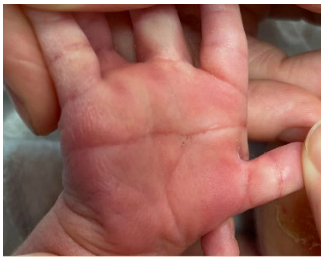

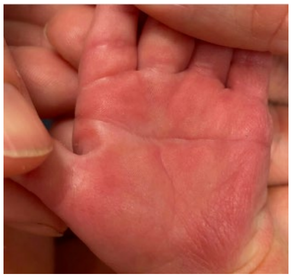

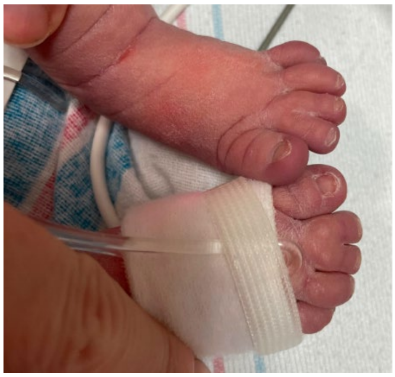

Add case 4, images show single palmar creases and 2,3 toe syndactyly.

GestaltMatcher analysis of facial images of Individuals 1-6

Introduction

Total potential novel syndrome associated with prenatal fentanyl exposure (new syndrome) images used in analysis: 6

Total unaffected images used in analysis: 11

Total Smith-Lemli-Opitz (SLO) syndrome images used in analysis: 10

Total fetal alcohol syndrome (FAS) images used in analysis: 11

Each image is translated to a vector of numbers that represents that image in the Clinical Face Phenotype Space.
Using these image numerical representations, we can calculate the syndromic similarity between them.

1. **Two dimensional representation of the distribution within the Clinical Face Phenotype Space**

Circles represent the 1SD range around the center for each group.

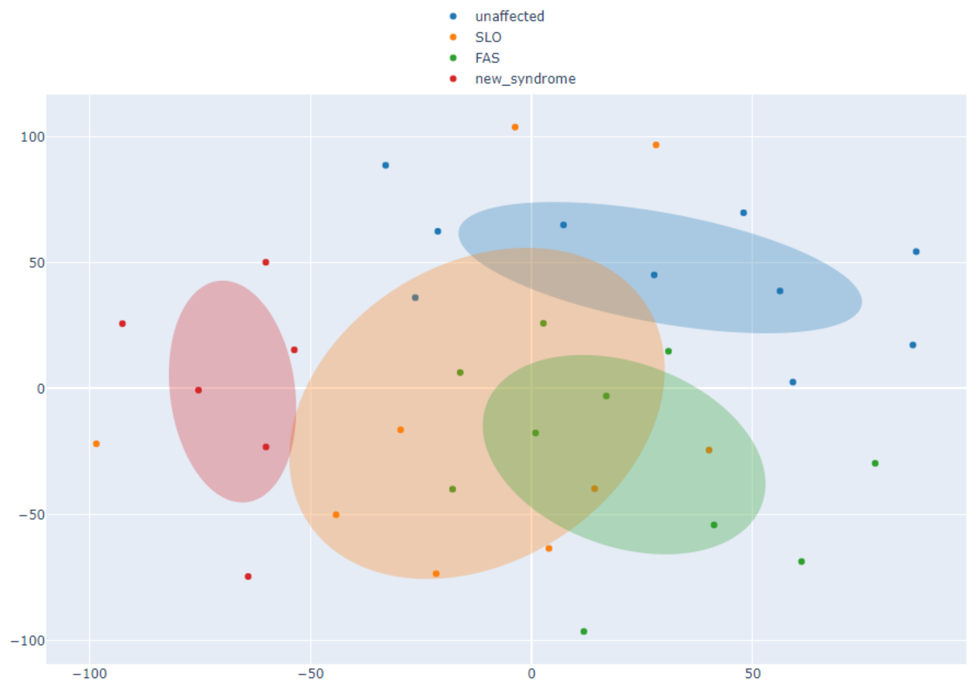

With the 2D TSNE visualization , we can see that the new syndrome seems to cluster nicely, although we still see some overlap between the confidence ellipses (considering 1 standard deviation around each cluster center).
However, this overlap seems to happen between all groups.

2. Intra-group similarity analysis

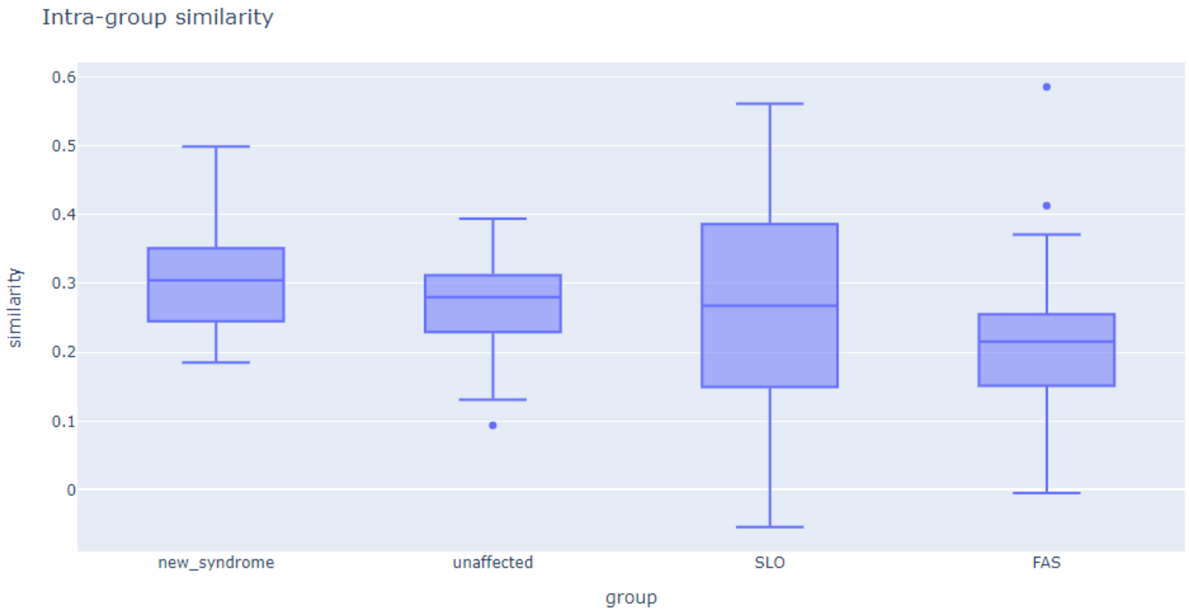

First, we **calculate the similarity between every image pair within each group.**

This is mainly a **sanity check** to see if the distributions look good, and if they are similar within each of the different groups.

We can see that the intra-group similarity distribution is similar between all groups.

3. Inter-group similarity analysis

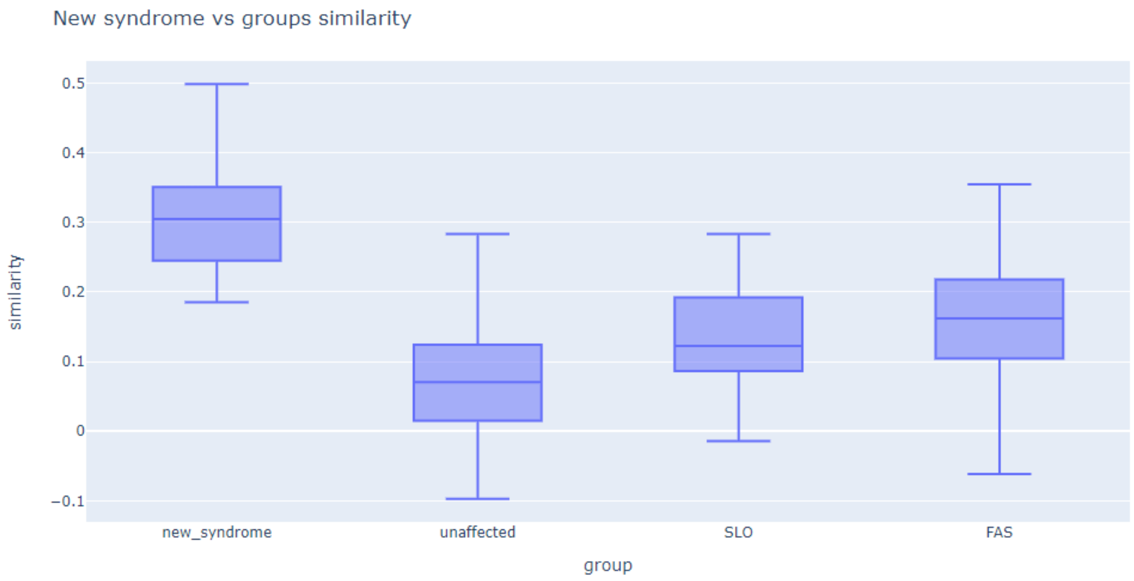

Next, we **calculate the similarity between every image vector of the new syndrome and every image vector of each group (including the new syndrome).**

Here we can see a **trend where the similarity within the new syndrome images is higher than the similarity between the new syndrome images compared to the images in other groups.**

Lets follow up with **statistical tests to check for significance.**

4. Statistical comparison between groups- checking for statistical significance

Assumptions checks

Are the samples independent?

True - no individual has data in more than one group.

Do the group samples have a normal distribution?

To assess this we'll use the **Shapiro-Wilk test**. It tests the null hypothesis that the data was drawn from a normal distribution (i.e. if the p-value > 0.05 we can reject the null hypothesis and the distribution is normal).

Do the group samples have equal variance?

To assess this we use the **Levene's test of homogeneity of variances**. The Levene test tests the null hypothesis that all input samples are from populations with equal variances (i.e. if the p-value > 0.05 the populations have equal variance).

Do the group samples have no outliers?

To assess this we use the **Grubb's test**. The test statistic corresponds to a p-value that represents the likelihood of seeing that outlier assuming the underlying data is Gaussian.

	group	target_group	ShapiroWilk_statistic	ShapiroWilk_pvalue	normal_distribution	Levene_statistic	Levene_pvalue	equal_variance	no_outliers
**0**	new_syndrome	new_syndrome	0.948514	0.501361	True	0.000000	1.000000	True	True
**1**	unaffected	new_syndrome	0.979921	0.425212	True	0.175340	0.676639	True	True
**2**	SLO	new_syndrome	0.974009	0.287611	True	1.480889	0.227905	True	True
**3**	FAS	new_syndrome	0.987614	0.803114	True	0.090142	0.764850	True	True

We can see that **all the group samples** have **normal distributions**, **equal variance** and **no outliers**. Therefore, we can use **parametric tests.**

One-way ANOVA

To determine if at least one group mean (or median) is significantly different from the others, and considering the above assumptions are correct, we can use the **One-way ANOVA test**.

T-Test comparison between pairs of groups

If the groups are different, we can do **follow-up multiple comparison tests, to determine exactly between which groups does the difference occur.** Considering the above assumptions checks results, we can do a **T-Test between each pair of groups**.

When implementing a large number of statistical tests in the same experiment, **the more tests we do, the higher probability of obtaining at least one test with statistical significance**.

The **Family-Wise Error Rate is the probability of making one or more false discoveries by running multiple comparisons**.

Therefore, **we must apply multiple comparison correction to prevent this problem.**

Based on our previous discussion, the **Bonferroni correction** was applied.

	test_name	groups	statistic	p-value	bonferroni_corrected_p-value
**0**	One-Way ANOVA	new_syndrome, unaffected, SLO, FAS	40.84795	0.0	NaN
**1**	Independent T-Test	new_syndrome, unaffected	9.96442	0.0	0.0
**2**	Independent T-Test	new_syndrome, SLO	8.33641	0.0	0.0
**3**	Independent T-Test	new_syndrome, FAS	6.36500	0.0	0.0

The **ANOVA results** indicate that **the 4 groups are statistically different.**

Following up with **comparison tests between the new syndrome group and the individual groups**, we can see that the difference between the new syndrome and all groups is **significant**, **even after applying the Bonferroni correction**.

All p-values are $p<0.00001$, indicating **highly statistically significant** results.

Shepard's criteria for teratogenicity in humans applied to the novel syndrome associated with prenatal fentanyl exposure

Criterion #	Criterion	Evidence related to novel syndrome associated with prenatal fentanyl exposure	Criterion met?
1	Proven exposure to the agent at one or more critical times during prenatal development	Exposure during entire pregnancy, including early pregnancy, admitted or highly suspected	yes
2	Consistent findings by $\geq$2 high-quality epidemiologic studies, with control of confounding factors, sufficient numbers, exclusion of positive and negative bias factors, prospective studies if possible, and relative risk $\geq$6	This information is not available yet.	no
3	Careful delineation of clinical cases; a specific defect or syndrome, if present, is very helpful	The individuals show a distinctive syndrome as delineated in this clinical report.	yes
4	Rare environmental exposure that is associated with a rare defect	Opioid use disorder is not common, though increasing. The described syndrome, resembling Smith-Lemli-Opitz syndrome and having a distinctive facial phenotype, is uncommon.	yes
5	Teratogenicity in experimental animals, important but not essential	No comparable phenotype has been reported in an animal model. Other opioids affect offspring in animal models.[1]	no
6	Association should make biological sense	Fentanyl is efficiently transferred across the placenta in early gestation.[2,3] Fentanyl transfer is not affected by simultaneous presence of heroin.[4] Other opioids have been associated with an increased risk for congenital anomalies, including cleft palate and talipes equinovarus.[5,6]	yes
7	Proof in an experimental system that the agent acts in an unaltered state	Fentanyl is the active substance	yes

The criteria listed here were proposed by Shepard.[7] Criteria 1, 2 and 3 or criteria 1 ,3 and 4 are considered essential, whereas criteria 5, 6 and 7 are helpful but not essential.

This table was adapted from Table 1 provided in Rasmussen et al., 2016.[8]

Bradford Hill criteria for evidence of causation applied to the novel syndrome associated with prenatal fentanyl exposure

Criterion	Evidence	Criterion met?
Strength of association	The additional cases reported here were quickly identified, however the total number of cases is small	yes
Consistency	Identification of the additional cases from outside institutions supports consistency of the phenotype associated with prenatal fentanyl exposure	yes
Specificity	This combination of physical findings with early biochemical findings of abnormal cholesterol metabolism in the absence of a diagnosis of Smith-Lemli-Opitz syndrome is specific; the facial phenotype is specific	yes
Temporality	The current fentanyl use epidemic supports temporality for a newly recognized syndrome associated with prenatal fentanyl exposure	yes
Biologic gradient	Dosage measurement and documentation is challenging for illegal use, but prospective information collected for affected pregnancies may identify a gradient and a spectrum disorder	no
Plausibility	Fentanyl is efficiently transferred across the human placenta in early gestation.[2,3] Fentanyl transfer is not affected by simultaneous presence of heroin.[4] Other opioids have been associated with an increased risk for congenital anomalies, including cleft palate and talipes equinovarus.[5,6]	yes
Coherence	Congenital anomalies resemble Smith-Lemli-Opitz syndrome and abnormal cholesterol metabolism is found in the early neonatal period. Other opioids have been associated with an increased risk for congenital anomalies, including cleft palate and talipes equinovarus.[5,6]	yes
Experiment	No animal model recapitulating the novel syndrome associated with prenatal fentanyl exposure in humans exists to our knowledge.	no
Analogy	Alcohol use disorder is associated with the fetal alcohol syndrome and fetal alcohol spectrum disorder. This condition is analogous as a use disorder with an effect on some, but not all, children born after prenatal exposure.	yes

This table was adapted from Table 2 provided in Rasmussen et al., 2016.[8] Bradford Hill criteria were originally delineated to provide guidance supporting causality, no defined minimum score has been postulated, rather each criterion should be considered.[9]

References

1. Byrnes EM and Vassoler FM. "Modeling prenatal opioid exposure in animals: Current findings and future directions." Frontiers in Neuroendocrinology 51 (2017): 1-13.

2. Shannon C, Jauniaux E, Gulbis B, Thiry P, Sitham M, Bromley L. Placental transfer of fentanyl in early human pregnancy. Hum Reprod. 1998 Aug;13(8):2317-20. doi: 10.1093/humrep/13.8.2317. PMID: 9756318.
3. Cooper J, Jauniaux E, Gulbis B, Quick D, Bromley L. Placental transfer of fentanyl in early human pregnancy and its detection in fetal brain. Br J Anaesth. 1999 Jun;82(6):929-31. doi: 10.1093/bja/82.6.929. PMID: 10562792.
4. Mortensen NP, Caffaro MM, Snyder RW, Yueh YL, Fennell TR. Placental trophoblast transfer of opioids following exposures to individual or mixtures of opioids in vitro. Exp Biol Med (Maywood). 2019 Jul;244(10):846-849. doi: 10.1177/1535370219851109. Epub 2019 May 15. PMID: 31091988; PMCID: PMC6643195.
5. Yazdy MM, Desai RJ, Brogly SB. Prescription Opioids in Pregnancy and Birth Outcomes: A Review of the Literature. J Pediatr Genet. 2015 Apr 1;4(2):56-70. doi: 10.1055/s-0035-1556740. PMID: 26998394.
6. Lind JN, Interrante JD, Ailes EC, Gilboa SM, Khan S, Frey MT, Dawson AL, Honein MA, Dowling NF, Razzaghi H, Creanga AA, Broussard CS. Maternal Use of Opioids During Pregnancy and Congenital Malformations: A Systematic Review. Pediatrics. 2017 Jun;139(6):e20164131. doi: 10.1542/peds.2016-4131. PMID: 28562278.
7. Shepard TH. 1994. “Proof” of human teratogenicity. Teratology 50:97-98
8. Rasmussen SA, Jamieson DJ, Honein MA, Petersen LR. Zika Virus and Birth Defects--Reviewing the Evidence for Causality. N Engl J Med. 2016 May 19;374(20):1981-7. doi: 10.1056/NEJMsr1604338. Epub 2016 Apr 13. PMID: 27074377.
9. Hill AB. The environment and disease: association or causation? Proc R Soc Med 1965;58:295-300
